# Current Advances in Assessment of Dog’s Emotions, Facial Expressions, and Their Use for Clinical Recognition of Pain

**DOI:** 10.3390/ani11113334

**Published:** 2021-11-22

**Authors:** Daniel Mota-Rojas, Míriam Marcet-Rius, Asahi Ogi, Ismael Hernández-Ávalos, Chiara Mariti, Julio Martínez-Burnes, Patricia Mora-Medina, Alejandro Casas, Adriana Domínguez, Brenda Reyes, Angelo Gazzano

**Affiliations:** 1 Neurophysiology of Pain, Behavior and Animal Welfare Assessment, DPAA, Universidad Autónoma Metropolitana (UAM), Mexico City 04960, Mexico; ale0164g@hotmail.com (A.C.); mvz.freena@gmail.com (A.D.); breyess_20@yahoo.com.mx (B.R.); 2 Animal Behaviour and Welfare Department, IRSEA (Research Institute in Semiochemistry and Applied Ethology), Quartier Salignan, 84400 Apt, France; m.marcet@group-irsea.com; 3 Department of Veterinary Sciences, University of Pisa, 56124 Pisa, Italy; asahi.ogi@vet.unipi.it (A.O.); chiara.mariti@unipi.it (C.M.); angelo.gazzano@unipi.it (A.G.); 4 Department of Biological Sciences, Clinical Pharmacology and Veterinary Anaesthesia, FESC, Universidad Nacional Autónoma de México (UNAM), Cuautitlán Izcalli 54714, Mexico; mvziha@hotmail.com; 5 Animal Health Group, Facultad de Medicina Veterinaria y Zootecnia, Universidad Autónoma de Tamaulipas, Victoria City 87000, Mexico; jmburnes@docentes.uat.edu.mx; 6 Department of Livestock Science, FESC, Universidad Nacional Autónoma de México (UNAM), Cuautitlán Izcalli 54714, Mexico; mormed2001@yahoo.com.mx

**Keywords:** animal welfare, emotions, human-dog interaction, pain, positive and negative stimuli

## Abstract

**Simple Summary:**

In several species, facial expressions have been associated with positive and negative emotions to communicate their mental state. In dogs, the interpretation of these muscle movements is relevant because of their close bond with humans. Currently, there is a discussion about whether facial expressions in domestic dogs can communicate emotions or are simply the result of mimicry and emotional contagion. This article will discuss the available literature on dogs’ facial expressions, anatomy and neurophysiology, and their association with emotions and adverse events such as pain. In this species, it is a challenge to identify and associate both factors due to domestication. This review aims to provide scientific support and understanding of facial expression in dogs as a clinical ethological tool.

**Abstract:**

Animals’ facial expressions are involuntary responses that serve to communicate the emotions that individuals feel. Due to their close co-existence with humans, broad attention has been given to identifying these expressions in certain species, especially dogs. This review aims to analyze and discuss the advances in identifying the facial expressions of domestic dogs and their clinical utility in recognizing pain as a method to improve daily practice and, in an accessible and effective way, assess the health outcome of dogs. This study focuses on aspects related to the anatomy and physiology of facial expressions in dogs, their emotions, and evaluations of their eyebrows, eyes, lips, and ear positions as changes that reflect pain or nociception. In this regard, research has found that dogs have anatomical configurations that allow them to generate changes in their expressions that similar canids—wolves, for example—cannot produce. Additionally, dogs can perceive emotions similar to those of their human tutors due to close human-animal interaction. This phenomenon—called “emotional contagion”—is triggered precisely by the dog’s capacity to identify their owners’ gestures and then react by emitting responses with either similar or opposed expressions that correspond to positive or negative stimuli, respectively. In conclusion, facial expressions are essential to maintaining social interaction between dogs and other species, as in their bond with humans. Moreover, this provides valuable information on emotions and the perception of pain, so in dogs, they can serve as valuable elements for recognizing and evaluating pain in clinical settings.

## 1. Introduction

Identifying facial expressions in animals has been relevant [1,2] since Darwin [3] stated that non-human animals can create innate expressions adaptable to each species. However, its purpose is still debatable. Whether they function as a non-verbal language to maintain social structure or to convey emotional states (neurophysiological changes associated with the recognition of pleasant and unpleasant emotions), in this sense, both emotional states and facial expressions require the integration of peripheral, autonomic, endocrine, and muscular responses, which involve the activation of various brain structures (i.e., the amygdala, hypothalamus, and brainstem) [4,5,6,7]. The understanding of the interaction between these responses and its neural pathways has led to studying facial expressions as a way to assess animal welfare through the identification of pain or stressful states in horses [8,9], sheep [10,11], laboratory animals [2], cows [12] and pigs [13,14,15,16].

In contrast, evolutionary and anatomical adaptations have been reported in domestic canines [17]. An example of these changes is raising the eyebrow, conferred by facial muscles only present in the dog, compared to wolves. These characteristics bestow a childish look or paedomorphic traits to this species [18].

The close relationship that dogs have with humans and the effects of domestication [19] has led domestic canines to develop the ability to detect, distinguish and respond to conspecific gestures [20,21,22]. Likewise, it has contributed to developing communication skills and interpreting their emotions, where emotions describe an internal state modulated by the central nervous system, in which physiological, behavioral, and cognitive mechanisms develop in response to a stimulus or event. Additionally, this phenomenon is considered as a form of empathy towards other individuals in their social circle [23]. The above supports the theory that facial expression plays an emotional role where identifying these visual signals is a means of emotional communication [24]. The idea can also be reinforced with the so-called “emotional contagion”, a process in which animals learn to identify expressions from the same or other species and react with a similar pattern [22].

In this way, research regarding this topic has associated lip or eyebrow movements with positive and negative emotional states (known as the state caused by emotions such as fear, pain, or disgust) during heterospecific relationships with humans, and these can provide information about the emotions perceived by dogs [25]. Given this scenario, the evaluation of facial expressions has a relevant clinical value for pain diagnosis [26] and can be used together with current pain assessments and recognition [27]. As mentioned by Häger et al. [11], conscious perception of pain is represented by a change in facial expressions, such as ears flattening and tension in the muscles of the nose, mouth, and the orbital region [28,29]. Identifying these changes has been proposed as an alternative to assess the degree of pain through grimace scales based on the facial expression of cats [30,31], pigs [13], mice [32], and rats [33].

Thus, understanding the different processes that involve animals’ emotions and how to assess them through facial expressions will surely be a valuable approach in medical fields of study, such as neuroscience, psychopharmacology, animal behavior, algology, and the science of animal welfare [34]. This review aims to discuss and analyze the current advances in identifying facial expressions of domestic dogs and their clinical utility to recognize emotions, including pain, as a method to improve daily practice and, in an accessible and effective way, assess dogs’ health outcomes.

## 2. Methodology

**Figure 1:**
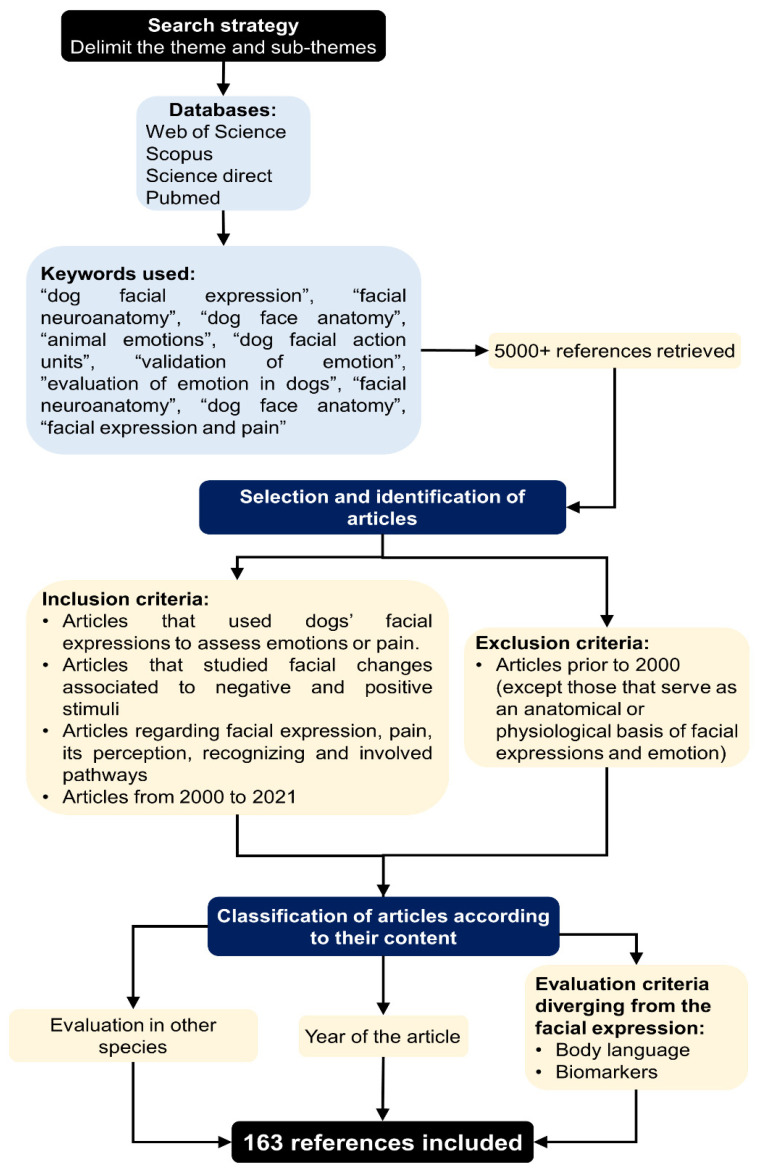
Review methodology.

A comprehensive literature review was conducted using *Web* of Science, Scopus, Science Direct, and PubMed. The keywords applied were related to “dog facial expression”, “animal emotions”, “dog facial action units”, “evaluation of emotions in dogs”, “validation of emotions”, “facial neuroanatomy”, “dog face anatomy”, and “facial expression and pain”. The inclusion criteria were articles published from 2000 to 2021, articles related to evaluating facial expressions in dogs and their emotions, the association between facial changes and pain perception, and pain conduction pathways and methods of identification and clinical recognition of pain. The exclusion criteria were articles related to other species and papers published before the year 2000 (except those that serve as a basis for understanding emotions or basic canine anatomy). Figure 1 describes the overall methodology for this review. 

## 3. Are Facial Expressions Involuntary and Emotional or Do They Have a Real Communication Purpose?

Darwin [3] argued that nonhuman animals communicate emotions through facial expressions. From the human perspective, canine facial expressions are considered a non-verbal communication tool not linked to the emotional concept [1]. This recent interpretation contrasts with the classical one, establishing that facial changes transmit information on six primary affective states: fear, happiness, anger, sadness, neutrality, and pain [35], which trigger specific responses, depending on the valence of the perceived emotion [36]. In animals, it has been suggested that facial expressions serve as a means of communication of emotional states [5]. Additionally, they also communicate sensations and intentions [37] to avoid predation and achieve a superior hierarchy [38].

**Figure 2:**
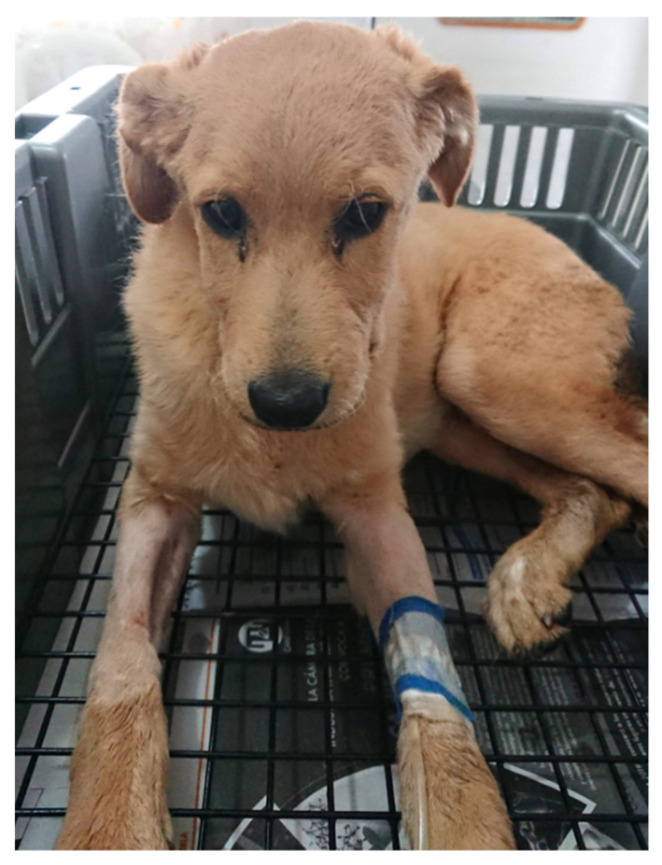
Characteristics of an infant-like, or paedomorphic face. A 3-month-old Golden Retriever puppy with gastrointestinal disease. The image shows the broad forehead and large eyes suggestive of sadness, two traits that may also be associated with chronic pain [39].

In dogs, the domestication process has changed some facial features that confer a paedomorphic or child-like appearance (Figure 2). These characteristics lead to feasible analogies with humans to study the meaning of facial expressions in dogs [21].

The study of facial expressions as a system of communication between conspecifics has been primarily investigated in dogs. Eyebrow and ear movements are used in dogs regularly to identify and communicate aversive states (referred to as an event caused by a noxious stimulus such as pain, distress, or any negative emotion), for example, flattening the ears or modifying the opening of the eyes during challenging stimuli [40]. In the same way, the movement of the lips, such as drawing forward the short lip and retraction of the labial commissure (in the long lip), communicate menacing states proportionally to the degree or intensity of the animal’s reaction [41,42].

However, recent evidence has shown that facial expressions may not have an emotional role. Kaminski et al. [43] evaluated 24 dogs of different breeds to assess whether canine facial expressions are subject to the effects of the presence of humans or a positive stimulus such as food. The facial movements were greater in dogs when the person was attentive than when he or she was not paying attention. Moreover, facial movements were not affected when a non-social stimulus such as food was presented. The authors concluded that canine facial expressions are influenced by the presence of humans and their level of attention. These findings suggest a communicative function, rather than emotional, that can serve as a nonverbal language to express aversive responses such as pain [44].

In a study by Bremhorst et al. [45], 21 Labrador X Labrador retrievers were exposed to two scenarios to assess facial changes while waiting for a reward with the presence or absence of people (social and non-social context, respectively). In this work, there was a significant difference in the frequency of movement of the inner brow raiser, which was more frequent during the non-social stage (*p* < 0.00001, F = 24.62). This confronts the idea that facial expressions are produced by perceiving emotions, like ear and lip movements in negative conditions [46].

Another study by Bloom and Friedman [25] classified the facial expressions of dogs through photographs analyzed by humans (including experts in canine behavior, non-experts, and people who had lived with dogs for years) who identified facial movements related to happiness, sadness, surprise, disgust, anger, fear, and neutrality. Despite the results obtained, the study concluded that further research is required because the human interpretation of animal expression and emotion is highly subjective and can lead to mistakes. The lack of awareness of the relationship between facial movements, behavior and a specific emotional state can be a factor that contributes to this effect, leading to inaccurate interpretation of the animal’s emotions. Therefore, it has been suggested that previous experience or knowledge about animal ethology influences the ability to recognize facial expressions. This subject will be addressed in detail later. Thus, the term “emotion” is a mental process that triggers reactions directed to specific internal and external stimuli which in animals is still controversial, since it is widely believed that emotions carry a subjective component that only humans can verbally express [47]. However, other authors differ and propose that emotions are physiological responses translated into corporal, vocal, and facial changes [48,49] that must be incorporated in future studies of animal emotion and behavior [5].

## 4. The Anatomy of Facial Expressions in Dogs

Currently, it is known that facial expressions are fundamental for the evolution and adaptation of species to their social environment and represent coordinated physiological and behavioral responses [36]. The standardized Facial Coding System (FACS) in animals is a detailed tool that identifies muscle movements using the so-called facial action units. These units identify and classify emotional states as neutral, fear, anger, and pleasure [25,50] based on the position and movement of the muscles. Movements such as the flattening of the ears, caused by the *inferior adductor auris, frontoscutilaris,* and the *retractor anguli occuli lateralis* muscles [46], as well as the nose wrinkles caused by lifting the upper lip due to the contraction of the *levator nasiolabialis*, as well as the *levator labii maxillaris* and *caninus*, are muscle movements associated with the perception of negative stimuli such as pain or fear in other species [51].

The facial nerve innervates and regulates the movement of the face through the cutaneous muscle of the neck (platysma), the zygomaticus, the buccinator, and the mentalis. This cranial nerve has both motor and sensory fibers afferents from the parasympathetic nervous system, regulated by the peripheral nervous system [52,53].

The facial nerve emerges from the cranial bone tissue of the stylomastoid foramen, located between the mastoid and styloid processes of the temporal bone. It innervates the occipital, posterior belly of the digastric, and stylohyoid muscles [54]. The facial nerve also leaves the skull and passes superficially to the parotid gland to originate its terminal branches in the cervical limits (temporal, zygomatic, buccal, marginal of the mandible, and cervical). These branches finally disperse and innervate the muscles of facial expression or facial mimicry [52,55].

The anatomy of the dog’s face has a series of muscles that produce specific movements recognized in this species. For example, the platysma muscle is composed of longitudinally arranged fibers that allow the caudal retraction of the labial commissure, described as lip tightener (AU24) by the Dog Facial Coding System (DogFACS) [56]. This division includes the upper and lower muscles. Among the superiors, *the frontalis, retractor anguli occuli lateralis, levator anguli occuli medialis,* and *orbicularis occuli* allow movements such as a brow lift (AU101) and eyelid closure (AU 143) [20]. In the lower muscle group, the digastric muscle in its posterior belly, the *buccinator*, the *orbicularis oris, zygomaticus, mentalis, caninus, levator labii maxillaris*, and *levator nasiolabialis* originate movements in the ventral edge of the jaw to push it down (AU25), open the dog’s mouth (AU17), deepen the nasolabial fold (AU11), or tighten the corners of the lips (AU27) [57,58,59]. Burrows et al. [60] mention that the marked anatomical differences in the faces of canids hinder reading their facial expressions because dogs have more than 20 facial movements; this is in comparison with other species, such as the chimpanzee or the cat, which only present 14 and 15 actions, respectively [61,62].

All canids have the same facial muscles, but beyond this anatomical similarity, it is essential to understand that the close co-existence of dogs with humans has developed some specific changes in dogs and breeds compared to wolves. Facial expressions in dogs allow them to manifest certain convenient gestures to maintain social structure [63]. One difference in dogs is the musculature responsible for eye movements, with significant development of the AU101 muscle. This muscle is also called the *levator anguli occuli medialis* (LAOM), or the interior elevator of the eyebrow. One of its functions is to give the impression of large eyes [18]. When it contracts, it enhances visual contact and confers a child-like appearance or paedomorphic features [18,64].

**Figure 3:**
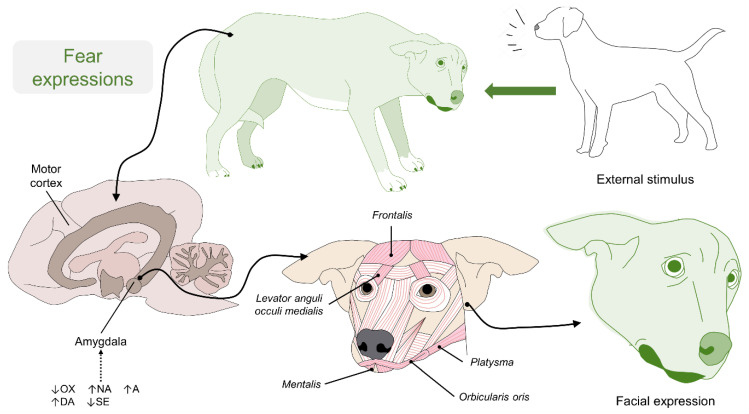
Neurobiology of facial expression in dogs. During a threat exposure, such as another dog, unfamiliar person, or a dispute over territory, the neural mechanism in the amygdala reacts to catecholamine secretion (A and NA). The catecholamines stimulate the motor cortex and its efferent fibers to modify a facial expression. Movements such as flattening the ears to the side (EAD105) and lifting the upper eyelids to have a wider field of vision (AU101) are characteristic of an expression of fear. OXT: oxytocin; NA: noradrenaline; A: adrenaline; DA: dopamine; SE: serotonin.

In the wolf, in contrast, the LAOM is small and surrounded by connective tissue, so the difference in movement in the AU101 region is greater in dogs [63]. This specific feature of the eyebrows in dogs is associated with dog-human interactions, which leads the latter to prefer certain breeds [65] (Figure 3). In comparison, observations of Siberian dogs, close relatives of the wolf, show little expressiveness in the eyes, because their muscular fibers are scarce and surrounded by large amounts of connective tissue, which impedes eyebrow movement. This internal muscular eyebrow movement in dogs is similar to human expressions of sadness, though it has also been associated with pain or malaise. It is worth mentioning that dogs developed specific movements due to these anatomical characteristics [63].

An example of this is the elevator of the eyebrow, which enlarges the eyes. Humans tend to associate this appearance with tenderness and care-motivation [65,66]. Although it has not been possible to demonstrate whether this characteristic represents a selection or preference advantage in humans [56], it is suggested that it may influence the interpretation of the mental state (a sensory state triggered by the perception of a specific stimulus). Understanding the emotional and facial repertoire is necessary to avoid misinterpretation of these movements, which are often mistaken as negative, such as sadness [67].

Another aspect to emphasize is that facial expressions involve more than one muscle. For example, the frontalis muscle, located in the frontal region between the scutiform cartilage and the upper eyelid, contracts in expressions of surprise and causes movement of the frontal region skin and the upper eyelid [68].

## 5. The Physiology of Facial Expressions

Animals’ facial expressions have been linked to manifestations of emotions. Studies have determined that triggering a response to an external stimulus requires, first, decodifying that stimulus [69,70]. The response is then performed by the central nervous system (SNC), specifically by activation of the limbic system (the ventral and dorsal regions of the cingulate gyrus, prefrontal cortex, ventral striatum, dorsomedial nucleus of the thalamus and amygdala). This network is responsible for identifying motivations and processing emotions, while the amygdala generates emotions and the associated facial expressions [71]. It is also involved in emotional memory and the recognition of non-facial external stimuli, including stimuli of an auditory, olfactory or gustatory nature [72]. The communication between the amygdala and other structures of the limbic system is mediated by the secretion of neurotransmitters, neuropeptides, and hormones. These neurochemicals are involved in the presence of emotions such as fear, anxiety, and happiness, in which particularly high concentrations of catecholamines such as dopamine (DA), noradrenaline (NA), and adrenaline (A) can be found [73,74].

The release of oxytocin (OXT) facilitates emotional memory, which promotes social learning [75]. Negative emotions are attributed to these neurotransmitters [76], while the presence of oxytocin at levels of 15 ng/mL can support the neurological process by compensating for social information and facilitating the feedback cognition of facial expressions, both happy and sad [75,77]. Therefore, neuroendocrine homeostasis between excitatory (DA, NA, and glutamate) and inhibitory substances (gamma-amino-butyric acid and serotonin (SE)) reflects a sympathetic or parasympathetic predominance, respectively, and acts together with the amygdala and the motor cortex (Figure 3).

In the first place, DA is associated with positive or reward-related emotions processed by cortical structures (medial prefrontal cortex, anterior cingulate, and olfactory cortex) and subcortical structures (striatum, amygdala, and hippocampus). These signals are integrated into the ventral tegmental area (VTA) and the *nucleus accumbens* [78].

**Figure 4:**
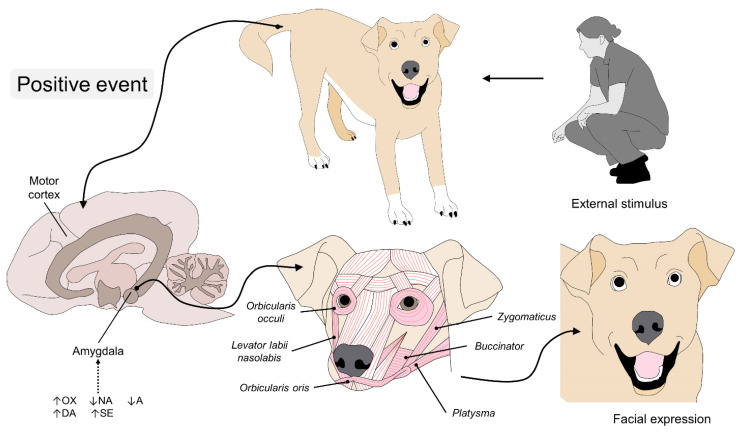
Endocrine and motor control of facial expression during pleasant emotions in canines. When the amygdala associates the presence of humans or conspecifics to a positive emotion, the levels of OXT, DA, and SE increase in response to the stimulus; this activates the facial units that enlarge the eyes and retract the muscles of the mouth to produce a simulated “smile”. OX: oxytocin; NA: noradrenaline; A: adrenaline; DA: dopamine; SE: serotonin.

In this sense, reduced concentrations of DA have been associated with pathologies such as chronic anxiety, while increases are related to events that trigger positive emotions, such as sports and training, together with other catecholamines (NA) and SE (Figure 4) [79]. Likewise, during techniques to reduce stress in dogs (e.g., petting), plasma levels of NA and SE (397.76 pg/mL and 542.75 ng/mL, respectively) have been associated with a relaxed state, a reaction that is also correlated to some lateralization of facial and body language [80]. SE also plays a role in the presentation of aggressiveness and the adjustment of facial expression, where low levels of SE in the prefrontal cortex are present in animals prone to aggressive behaviors [81].

On the other hand, OXT reduces amygdala activation in response to facial expressions and negative emotions like fear or rage by intensifying the connection between it and other regulators, namely the orbitofrontal cortex, cingulate gyrus, and temporal sulcus [82]. Studies in humans show that OXT fosters the capacity to infer the affective mental states of others based on subtle facial signals [83]. Low basal OXT levels have been associated with positive emotions in various species [84]. In dogs, Mitsui et al. [85] determined increased levels of urinary OXT in six Labrador Retriever dogs exposed to positive events such as eating, exercising and stroking (*p* < 0.05, *p* < 0.05, *p* < 0.01, respectively), which reflects relaxation, calm and security. Moreover, research has further ascertained that animals show facial expressions associated with emotions and that low basal oxytocin levels mediate these. In this context, Lansade et al. [86] evaluated the facial expressions of Welsh mares under two treatments of tactile stimulation (grooming): one traditional treatment did not consider the animals’ reactions but provoked avoidance, while the other treatment was directed towards regions that the mares considered positive or pleasant. Their work evaluated the horses’ facial expressions by observing the positions of the ears, eyelids, lips, and neck, among other gestural indicators. They determined that the cordial, pleasant grooming of the mares significantly increased expressions seen in the position of the neck, which rose to a medium height (*p* = 0.01), accompanied by semi-closed eyes (*p* = 0.01), lips contracted and extended laterally (*p* = 0.003), and the upper lip extended and immobile (*p* < 0.0001). These findings led them to conclude that the facial expressions were modified markedly in the presence of a low concentration of oxytocin compared to basal ones, suggesting that they could be considered positive emotions in horses. Their findings also reveal that oxytocin is an endocrinological marker of emotional well-being responses.

Increases in peripheral OXT after positive human-dog interaction have been shown [87,88], and, concurrently, the administration of exogenous OXT has also been shown to influence how dogs respond to human emotions and during positive interactions. Somppi et al. [89] evaluated 43 dogs of 16 different breeds. Gazing behavior and the diameter of the pupil were assessed to determine the emotion of the animals. Dogs receiving nasal OXT fixated more often at happy human faces than angry faces and had a larger pupil diameter. The authors attribute this response to the influence of OXT on attention and emotional arousal, which decreases vigilance reactions, diverts attention to positive events, and facilitates human-animal and animal-animal communication, as well as the mediation of social perception and emotional states in canines.

These studies confirm that the generation of facial expressions is due primarily to the decodification of external stimuli by the SNC through connections between the limbic system. Thus, this system is also responsible for responding to facial or non-facial stimuli and even positive or negative emotions, the former mediated by the release of hormones like oxytocin that mediate motor changes in facial muscles. In animals, whose limitation is being unable to self-report their mental state, it is essential to understand the neurobiological mechanisms involved in the perception and development of emotions [78]. However, despite these endocrinological alterations, in dogs, the question remains whether facial expression reflects emotions and serves as a communication tool or is the result of the manipulation and mimic of human gestures [90].

## 6. Emotions in the Dog

Anderson and Adolphs [91] define emotions as internal states of the CNS that generate physiological, behavioral, cognitive, and subjective responses to manifest distinct categories of states. In humans, emotions constitute a complex behavioral phenomenon involving multiple levels of neuronal and chemical integration [92]. These internal states result from the evolution of primary emotions (anger, fear, disgust, happiness, sadness, and surprise) and are associated with well-defined neuronal and neurophysiological chemicals that act as supporting factors to ensure the performance of specialized survival functions [35,93].

In general, gestures communicate emotional states and allow individuals to appraise social interactions [94]. Therefore, facial expression is an effective non-verbal information tool [95] to promote, create and facilitate social interactions between animals [96]. Consequently, facial expressions are one of the principal elements that allow individuals to identify the emotions expressed by others in a social group [19,97] and are the main pathway for transmitting the affective information that leads them to act in any given situation involving an emitter and a receiver of facial signals [98]. Even though it is still controversial to state that dogs use facial gestures to communicate their mental state, in a study conducted by Karl et al. [99], the response of 12 domestic dogs to positive social and non-social neutral stimuli was assessed using functional magnetic resonance imaging. The results showed that limbic areas such as the left amygdala, hypothalamus, and insula were activated during positive social interactions. The above shows the correlation between the neural response for the processing of emotions and the possible facial response, which would support the theory that dogs can transmit their emotional state using facial expressions. Besides, the personality and empathy of people have been correlated with their ability to interpret the emotions of animals. In this sense, it has been reported that empathic people can discriminate between positive and negative affective states [100]. However, independent of human perception, the possibility that dogs can transmit their emotional state with facial expressions cannot be denied.

Siniscalchi et al. [40] mention that domestic dogs use a broad range of facial regions to communicate their emotional condition; however, the orbital region plays a crucial role in interacting with conspecifics. For example, in stressful situations, the dog opens its eyes, exposes the white sclera, and flattens the ears (EAD105), gestures that indicate warning of an imminent threat [41,101]. In this context, Caeiro et al. [102] evaluated the response of the face of dogs and humans during the perception of different visual and auditory stimuli using the FACS system in both species. The authors observed that dogs emit facial movements depending on the perceived stimulus. In addition, they observed that the facial movements are different from those observed in humans during similar emotional states. Similar facial movements have been identified in dogs and humans, such as the eyebrow-raise or the upper lip raise (AU110). However, it may be inappropriate to consider that they have the same emotional valence. For example, a raised upper lip is often mistakenly interpreted as a smiling expression by children [103].

**Figure 5:**
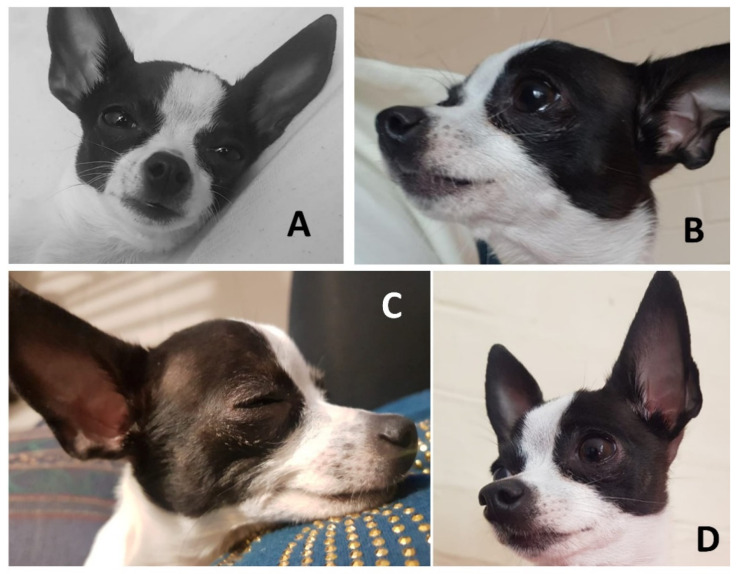
Facial expressions in dogs and their links to humans. Image of a one-year-old female Chihuahua showing marked changes in facial expressions upon recognizing similar emotions in her owner’s gestures. (**A**) Neutral. Relaxed expression; (**B**) Sadness. Broad forehead, large eyes, ears erect with caudal inclination, closed snout; (**C**) Pleasure. Tension in the labial commissure, eyes semi-closed with facial mimicking that simulates the form of a human smile; (**D**) Surprise. Eyebrows raised to show apparently larger, more expressive eyes, with ears raised and labial tension.

Similarly, Konok et al. [5] reported that humans attempting to describe dogs’ emotions have an anthropomorphic influence. Although relevant for animal welfare, humans tend to interpret the emotional states of animals in the same terms they use to perceive their own. However, as stated before, it is not appropriate to assume animal emotions based solely on human perception and anthropomorphization of human feelings or facial expressions. On the other hand, Bloom et al. [104] reported that 105 liberal arts students were able to successfully determine the emotional states of canines (joy, sadness, surprise, disgust, anger, fear, and neutral) using the DogFACS, which has proven to be an adequate tool to assess emotions and facial expressions [25] (Figure 5).

**Figure 6:**
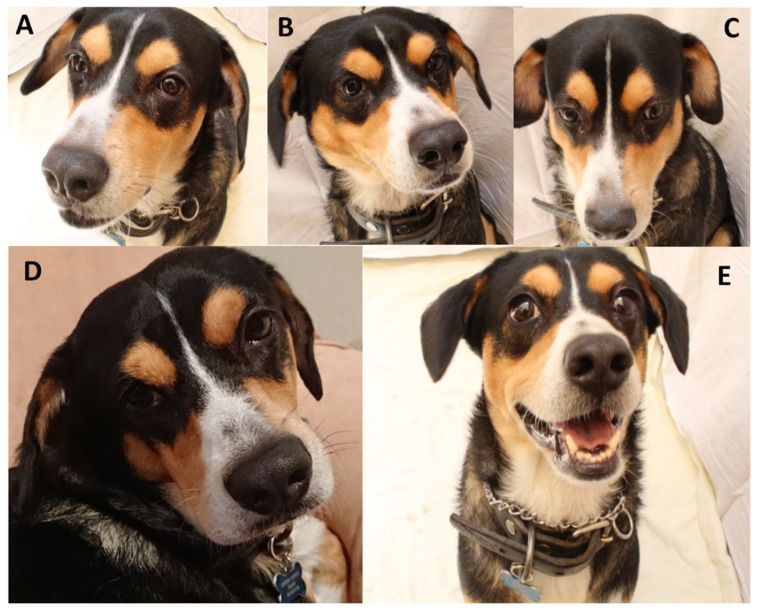
Changes in facial expression during exposure to various stimuli. (**A**). Neutral. No relation or expression; (**B**). Anger. Observations include a fixed gaze with ears at medium height or pulled back with flared nostrils and tensed cheeks; (**C**). Fear. The gaze has deviated with the ears pulled back, exposing the frontal region as a signal of submission; (**D**). Surprise. The eyebrows are inexpressive, perhaps with a slight raising of the medial section to give the appearance of large eyes, with the ears at medium height; (**E**). Pleasure. Eyebrows and ears are clearly raised with the mouth open and tension in the labial commissure.

Facial movements are also considered an adaptive response to threatening stimuli. However, their interpretation depends not only on conspecifics but also on the humans with whom dogs establish bonds [105]. Flint et al. [106] have evaluated the association between facial and body movements, a negative emotional response such as fear, and the reliability of the owner to identify these changes. Through videos, the accuracy of the owners of 735 dogs was evaluated to detect fear-related behaviors, focusing on the head, changes in posture, and facial and body language. The authors found that owners accurately identified the position of the ears (flattened ears (EAD015)) as a facial modification associated with fear with a sensitivity and specificity of 0.76 and 0.88, respectively. Other changes reported with a sensitivity and specificity above 0.75 were a low posture, lowered tail, hiding, escape attempts, panting, yawning, lip licking, and no eye contact. Additionally, based on the behaviors and postures identified by the owners, a training tool was developed to improve the recognition of fear behaviors. Although the differences after the training did not significantly influence the reliability of assessing fear in dogs, owners were more likely to recognize “mild to moderate fear” (OR: 1.01, 95% CI: 0.86, *p* = 0.881) based on the mentioned facial expressions such as the position of the ears and the other related behaviors. In contrast, as stated by Bremhorst et al. [47], when exposed to positive events (food and toys), 29 dogs of one breed showed erect ears (AED101) and blinking (AU143 + AU145). Under negative conditions (ignored by the owner), nose licking, drooping jaw (AU26), drooping lip (AU25), and flattened ears (EAD105) were predominant (Figure 6). Therefore, both studies show that recognizing these facial movements, particularly the position of the ears, could contribute significantly to the identification and interpretation of dogs’ emotional state, representing a wide field to study in the future.

**Figure 7:**
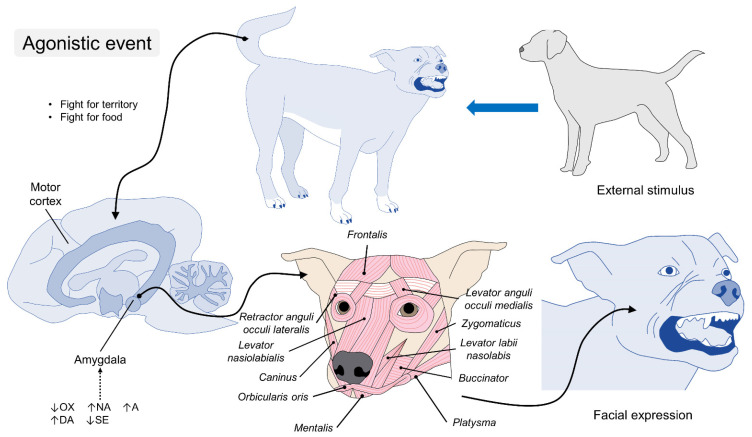
Facial expressions represent aggression. The perception of an adverse external stimulus (e.g., a person or another animal) triggers a neuroendocrine response with an elevation in catecholamine levels, together with a decrease in circulating levels of OXT, DA, and SE. When this negative emotion is recognized in the amygdala, its connections to the motor cortex and facial muscles (trough the facial nerve) cause the flattening of the ears (EAD105), lifting of the upper lip to bare their teeth (AU116), nostril opening (AD38), more visible white sclera (AU101), and vocalizations (AU34) as a sign of threat. OXT: oxytocin; NA: noradrenaline; A: adrenaline; DA: dopamine; SE: serotonin.

Since dogs are highly sociable animals with complex groups, understanding heterospecific emotions is of particular importance [107]. Racca et al. [108] demonstrated this in their study that assessed dogs’ capacity to discern facial changes based on visual stimuli and if such behavioral responses are influenced by the species observed (i.e., human vs. conspecific faces). Their study protocol involved seven dogs of different breeds in two phases. The first type of discrimination was determined using images of (i) a familiar visual stimulus and (ii) a novel one. They found that the dogs showed greater interest in the novel stimulus and could discriminate objects presented simultaneously. In the second stage, a simultaneous face discrimination test using images of humans vs. different breeds of dogs, the authors found that the time the dogs invested in observing the images averaged 4.2 s more for the new rostrums of both humans and conspecifics. Those authors concluded that canines could perform face discrimination regardless of species and modify their facial expression responses depending on the type of face shown. In a posterior study by the same authors, the ability of dogs to process facial expressions was compared to those of 4-year-old children, showing them a series of photos with emotional facial valence (positive, negative, or neutral). In this case, they videotaped the subjects’ facial expressions and eye movements. The authors found that the dogs spent less time observing the images (3.87 s vs. 4.51 s in children) and reported greater eye movement latency (1.21 s vs. 0.66 s in children). The comparison of the responses of the two groups detected a significant difference in the processing of the positive images, as opposite responses occurred concerning these emotions; one example of this was deviating the gaze, which was identified as the behavior that canines exhibit when distinguishing between happy and sad faces [109]. Both results confirm sensitivity to facial expressions such that dogs can interpret and react despite being individuals of different species, as in, for example, the gaze response to a threatening expression (Figure 7) [110]. A particular communication mechanism between dogs and humans has been observed, as stated by Ogura et al. [111]. The gazing behavior was evaluated in seven dogs of different breeds exposed to different situations: humans using hand signals, other dogs, and the presence of a cat. The dog paid more attention to the hand signals of humans but focused more on the face and body when looking at conspecifics and cats. This research raises a probable species-dependent communication difference.

For these reasons, facial expressions that reflect different emotional states may well prove to be an effective tool for (i) achieving non-verbal communication [95]; (ii) creating, fomenting, and facilitating social interaction among animals; (iii) constructing or improving successful social groups, as mentioned by Brudzynski [96], and the affiliative function of expressions that promotes contact and closeness of members to each other [84]; and (iv) providing relevant information that, together with other gestural signs, may modify the interpretation and evaluation of such communications [19,97]. In this regard, fear and anxiety refer to what we consider emotional responses to aversive stimuli. These emotions are adaptive for survival because they are triggered to avoid a perceived or anticipated threatening stimulus [105]. It has been established that those behavioral problems in dogs are probably associated with a close relationship to humans. Hence it is vital to avoid these states [112], as Mariti et al. demonstrated [107]. Those researchers further concluded that the dog owners did not require experience to identify these negative emotions accurately. Owners were able to identify the behaviors and expressions associated with aversive states without needing any training to identify them. This will be discussed in detail later.

In addition, the ability of dogs to read the gestures emitted by humans may be related to another phenomenon that occurs when individuals from social groups recognize certain expressions from the same species or others and respond with a similar or antagonistic pattern [104]. This process is called “emotional contagion.” The above has been reported in rats, animals that, after encountering conspecifics with pain, manifested pain gestures and behaviors without having any injury [113,114]. This suggests that animals can display a similar state after reading the facial language, although facial mimicry does not reflect the meaning of the inherent emotion on every occasion [115]. Using emotional contagion, researchers have sought to ascertain whether dogs can show interest in the emotions expressed by other individuals in their social circle. In this field, Katayama et al. [22] observed that the emotional contagion of humans towards dogs occurs more pronouncedly with women and is affected by the surroundings in which it occurs. They evaluated the response of 34 dogs towards their owners while they carried out a reading test with an audience, considered a stressful event for humans. They reported a correlation between the R intervals of the heartbeats present in dogs and humans (r^2^ = 0.74, *p* <0.01), and that in addition, the emotional contagion of humans towards the dog was influenced by the sex characteristics of the pet and the time of coexistence, since in females and in individuals who had a long time of coexistence with the owner, the emotional contagion was higher.

Although this phenomenon is a primitive form of empathy between individuals who share the same social environment, it does not necessarily involve a higher psychological function [23]. However, it is a process that improves the human-animal interaction, particularly in domestic canines who have adapted and refined mechanisms to live inside mixed-species groups [98].

As can be seen, comprehending the emotions and affects of heterospecifics is of great significance for domestic species, such as dogs [110]. Recognition of emotions, mimicry, and gestures is considered of greater importance than other forms of expression [115], though facial mimicry does not necessarily accurately reflect the meaning of the underlying emotion [112]. This phenomenon has also been identified in dogs because their ability to recognize certain expressions shown by individuals in their social circle—whether of the same or a different species, including humans—allows them to generate similar or opposing responses to those manifestations. Those responses take the form of facial expressions that reflect perceived states [102] (Figure 6).

Various emotional and behavioral problems described in dogs are attributed to their close interaction with humans and modern lifestyles [116]. These led Kurachi et al. [117] to state that behavioral problems in animals must consider their physical and emotional origins and the human-animal bond that can influence their welfare. To achieve an acceptable level of welfare, animals require positive events (e.g., pleasure) instead of only preventing negative ones (e.g., fear or anxiety) [4,118].

In this context, further research is required in dogs to evaluate positive emotional states and associate them with facial and body movements, as well as in other species, like in pigs [119], in sheep [120], and in horses [121], among others. The emotional state influences the behavior, communication, social bond, and cognitive functions of the group’s individuals [122]. Then, the emotions are expressions of the internal states of the CNS reflected in animals’ physiological and behavioral responses, which foster better interaction among the individuals that make up a social circle. Identifying these expressions is an additional, important indicator for recognizing the degree of welfare that animals experience.

## 7. Interpretation of Perception and Expression Interspecies

Currently, there is a growing interest in interpreting dogs’ emotions to enhance the welfare of both species: dogs and humans [39,123]. Flint et al. [106] asked a group of dog owners to distinguish expressions of fear in their pets to identify animal facial expressions linked to aversive stimuli. Using videos, they demonstrated the importance of several regions, especially the head, body posture, and facial expressions. The owners adequately recognized low posture, ears pulled back and tail between the legs or curled downwards as behavioral gestures that indicated concealment, attempts to flee, panting, yawning, lip-licking, and null visual contact. In this context, Bloom and Friedman [25] designed a study to ascertain people’s ability to identify facial expressions emitted by dogs in the presence of diverse stimuli and, on that basis, attempted to define such emotions as happiness, sadness, surprise, disgust, anger, and fear. They utilized positive and negative stimuli to provoke dogs, and they found that when their owners emitted positive stimuli, the dogs’ reactions included wide-open eyes, erect ears, and a fixed, forward gaze (Figure 6E). When the element of surprise was added, the dogs showed furrowing in the central region between their ears, a slightly inclined head, raised eyebrows, and the mouth closed or only slightly open. In contrast, when negative stimuli were shown, the dogs tended to bow the head and deviate their gaze laterally, accompanied by a differential raising of the two eyebrows, caudally inclined, erect ears, and the mouth closed or slightly open (Figure 6C). When given medication, the dogs inclined their ears towards the caudal region (EAD105), bared their teeth slightly (AU118), and showed a slight lowering of the nose (AD40) and eyebrows (Figure 6C).

**Figure 8:**
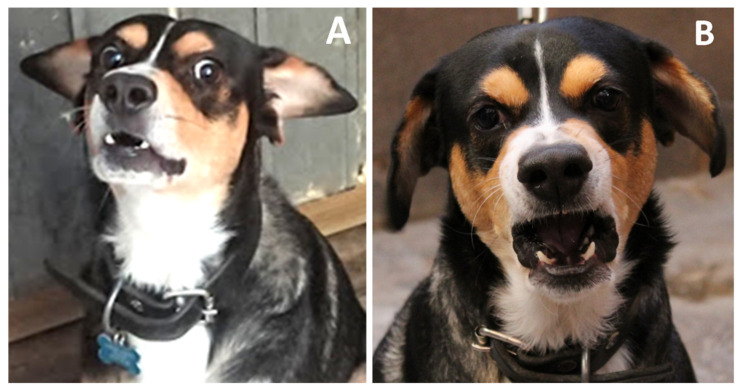
Facial expressions related to fear can provoke aggression. (**A**) Nose, sclera, and snout. Increased exposure of the sclera is caused by raised eyebrows and tension in the nasal region with flared nostrils, accompanied by an open mouth, raised upper lip, and exposed fangs. Additionally, dogs may emit vocalizations. (**B**) Ears and gaze. The positioning of the ears is commonly seen to be at a medium height concerning the frontal region; the animal’s gaze is fixed in the direction of the threatening stimulus.

When dogs are induced to feel fear through verbal communication, their ears are markedly erect; their eyes show a small display of the sclera, their gaze shifts from side to side, and the mouth is either slightly open or tightly shut (Figure 8A). Finally, upon hearing the negative stimulus, bad boy, “the dogs exponentially bared their teeth and fixed their gaze on the objective with eyebrows pointing towards the center, ears folded rearwards, and the mouth semi-open or open (Figure 8B).

In the same study, the expressions of the animals in the absence of a stimulus (neutral) were also evaluated. The resulting photos were like those taken after a positive stimulus—“good boy—, with a slight lateral inclination of the ears. In contrast, Racca et al. [109] determined that, in dogs, a neutral human facial expression is categorized as a negative emotion. They concluded that the human recognition and interpretation of dogs’ facial expressions could trigger a certain degree of expected behavior by the owner or handler and improve their interspecies interaction.

It is important to emphasize that no previous experience or training is necessary for people to identify emotions in dogs based on their facial expressions. Comparative studies have shown that the ability to interpret animal behaviors is influenced by the level of experience of the evaluator [124]. Lakestani et al. [125] compared the ability of children and adults to understand the friendly and aggressive behavior of dogs. In this study, young individuals could not discern friendly from aggressive behaviors (*p* < 0.001). This was attributed to children focusing more often on the face of the animal, leading to misinterpretation (x^2^ = 80.2, df = 1, *p* < 0.001). These results reinforce the idea that the experience and ability of the evaluator influence their ability to decipher facial expressions and body language of companion animals. However, a study by Tami and Gallagher [123], which compared observations of the body language and facial expressions of untrained people to those of individuals who had some knowledge of canine behavior (e.g., dog trainers or owners), demonstrated that both groups of evaluators found it challenging to distinguish between aggression and playfighting. For this reason, integrating facial expressions with other gestural signs could be a key to elaborating a comprehensive assessment of dogs in this regard.

Humans perceive raised eyebrows, characterized by an increase in the overall size of the orbital region, as a sign of sadness [21]. According to the description by Merola et al. [126], a backwards gaze seems to indicate a request for some object and to verify humans’ reaction to an ambiguous object. In this sense, people also confer an anthropomorphic sense to the gaze of animals, particularly the so-called “guilty look,” which is associated with this feeling of guilt by around 74% of people when observing said facial expression. This gesture is characterized by ethologists by an aversive gaze, lowered ears, constant licking, and a lowered head position. However, there is a difference between what humans interpret and the actual reason for the facial motor response. For example, in a study with 14 mongrels and purebred dogs, there was no difference in the prevalence of guilty gaze when the animals performed an acceptable behavior or not. The authors concluded that dogs respond with a guilty look when scolded, regardless of having infringed an instruction or not, which means that the animal does not understand that it has disobeyed and lacks a cognitive association [127].

In recent years, eye-tracking systems have become relevant to distinguish facial language associated with positive or negative stimuli [128]. In these studies, canines confer more attention to body language, unlike humans, who prioritize the grimaces of fellow humans and other animals. Correia-Caeiro et al. [129] evaluated the reaction of 129 humans and 92 dogs to videos with gestures of positive (happiness, pleasure) and negative (anger) emotions from both species. The included animals responded with several gestures to expressions of conspecifics and humans, with no evidence of facial mimicry towards the recordings. Interestingly, the action of the EAD102 (ear adductor) unit was more frequent when observing other dogs’ faces, which can be interpreted as a positive response to the same species [130,131].

Another element of facial expressions that needs evaluation in dogs involves the lips, since these function as messengers to other individuals that express aggressive intentions; that is, displays with lips retracted, the snout thrust forward (simulating smaller lips), with the mouth open. These expressions reflect a threat of a particular type or degree [132] (Figure 6B). Comparatively, between humans and dogs, Action Unit 27 (AU27), representing the wide opening of the mouth, and AU6, where the muscle around the eyes contract and the cheeks are raised as a “happy face,” are similar facial expressions in both species [129]. Similarly, lip-licking in dogs is understood as a behavior involved in identifying expressions of anger in humans [98].

Firnkes et al. [133], in addition, reported that a deviated gaze and lip-licking are associated with appeasement. Appeasement includes all those behaviors aimed to avoid confrontations with conspecifics and other species. The objective of their research was to examine manifestations of these signals towards unfamiliar people using a standardized behavioral test. Their findings suggest that a deviated gaze and lip-licking occur during appeasement and, more often, when dogs face situations involving threat or conflict. However, these signals decrease when the threat is greater and are replaced by behaviors indicative of submission (Figure 6C) or attempts to flee, defining submission as those behaviors used to avoid the threat represented by a dominant member of the same species. Those authors concluded that dogs seem to have the ability to employ distinct strategies according to the type of situation. Additionally, they were able to identify that lip-licking forms part of dogs’ greeting behavior towards humans, since socio-positive focuses were observed with greater frequency. For this reason, this gesticulation can play a crucial role in intra-specific communication by serving as a signal that expresses peaceful intentions.

Regarding evidence for the lateralization of facial movements in domestic canines, Nagasawa et al. [134] analyzed whether dogs could do this when encountering their owners. Lateralization of emotion is related to activating the right or left hemisphere following positive or negative emotion recognition in the brain. The goal was to determine whether lateralization was correlated to positive and non-positive social events (presence of the owner, or toys, respectively). The authors found that when the stimuli were of a social type, dogs showed greater laterality towards emotive stimuli by responding with a facial expression involving the eyebrows or ears of the left side, compared to the case of inanimate objects that they had to avoid, where they showed greater facial expression on the right side. Similarly, lateralization of the gaze has been reported and related to the ability of dogs to recognize emotions and activate the right or left cerebral hemisphere, a relevant characteristic in animals with frontal eyes, whose visual connection usually responds to activation of the contralateral hemisphere. For example, a gaze to the left indicates activity in the right hemisphere, associated with neutral human faces. Racca et al. [109] evaluated the reaction of 21 canines of various breeds to negative, neutral, and positive expressions of conspecifics. The results showed that the maintenance of a gaze to the left, in response to a negative expression, was greater (1.60 ± 0.20 s) compared to the gaze to the right (0.57 ± 0.09 s) and neutral and positive stimuli (1.26 ± 0.16 and 0.65 ± 0.11 s, respectively). This can be translated as a predominance of right hemisphere activity, as the right hemisphere is a structure involved in interpreting negative emotions. Moreover, a prevalence of head-turning to the left in animals exposed to anger or fear human gestures has been reported [132].

It is worth mentioning that there are current reports that define limitations in the assessment of the emotions through the eyebrows and lips in the dog. Some examples are a paedomorphic face, the type, length, and color of the hair, and the anatomical differences between breeds (e.g., the structure of the skull) [104]. Additionally, dark-colored spots on the face of canines, particularly in the eyebrows, tend to give an aggressive appearance and are considered another factor that can alter the perception of humans regarding the emotion of companion animals [132,135].

As can be seen, two critical regions to be considered when evaluating facial expressions in dogs are the position of the lips and eyebrows, since they allow us to distinguish between stimuli perceived as positive vs. negative. Dogs capture these stimuli to identify intentions such as threat or play, though they present a certain degree of difficulty for assessment because some may be similar and impede the ability to arrive at accurate interpretations of the emotion shown.

## 8. Changes in Facial Expressions Related to Pain

**Figure 9:**
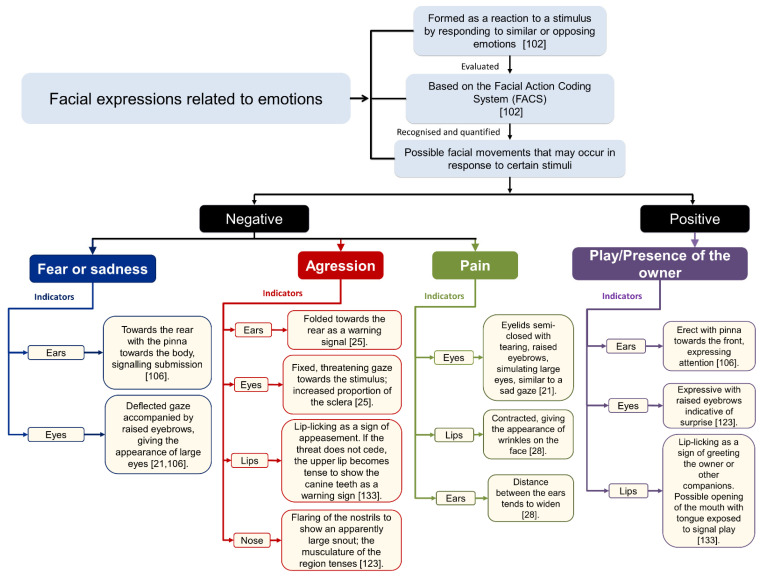
Facial expressions related to emotions [21,25,28,102,106,123,133].

The identification of facial expressions in animals offers an opportunity to assess, in a non-invasive way, the welfare of domestic species [16]. As stated by the International Association for the Study of Pain, pain is “an unpleasant sensory and emotional experience associated with, or resembling that associated with, actual or potential tissue damage” and has a connection with gestures and the degree of pain [136]. The current approach to pain assessment is based on the affective magnitude (e.g., “how does it make you feel?”). Therefore, facial expressions are an alternative to evaluate pain and differentiate this state from other emotional states such as fear, stress or anxiety (Figure 9) [26,137,138].

With the implementation of FACS, it has been possible to identify subjective states such as pain. The tension of the muscles of the face, lips, orbital region, and flattening of the ears are commonly recognized in animals [2,139,140,141,142,143,144,145,146,147,148,149,150]. This system has been adapted as a veterinary clinical tool (called Grimace Scales) to identify pain in species such as equines [142], chimpanzees [60], macaques [143], sheep [8], rabbits [144], ferrets [145] and rats [33].

**Figure 10:**
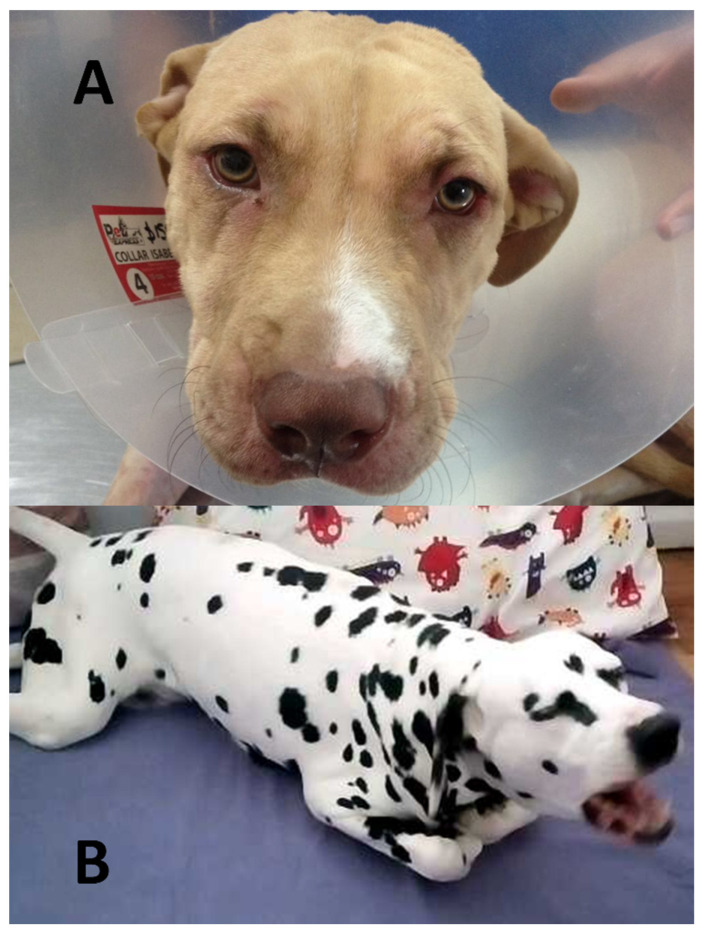
Expressions related to pain. (**A**). Mild pain. Expressions are characterized by a sustained raising of the eyebrows with displays of the frontal region of the head and lateral abduction of the ears, generally associated with a sad face, as represented in this image of a one-year-old female Pitbull, which shows a slight anaphylactic reaction. (**B**). Severe pain. Expressions are characterized by semi-closed eyelids, cheek tension, and vocalizations, as can be seen in the image of this two-year-old female Dalmatian with acute polyradiculoneuritis.

Behavioral evaluations of pain must consider several indicators. In dogs, Camps et al. [146] described the principal characteristics of aggression related to pain. They affirm that behavioral changes are the most common indicator of pain manifested by this species through such behaviors as aggression, fear, vocalizations, reduced interaction with conspecifics or family members, and altered postures or facial expressions, restlessness, and concealment (Figure 10).

In equines, facial characteristics such as asymmetric ears, the tension of orbital muscles, and the lips decrease their frequency of appearance during the administration of analgesics [147]; these characteristics are known as the “face of pain” [140]. On the other hand, in felines, observed facial movements positively correlated with the Feline Grimace Scale (r^2^ = 0.86), a proposed validated tool to evaluate nociception in cats [29].

In felines, the Feline Grimace Scale (FGS) has been implemented to identify acute pain using facial expressions, with an efficacy of up to 94% to differentiate between pain and pain-free cats. These scales have made it possible to identify that 85% of the facial changes observed during a painful event are concentrated in the ears, eyes, whiskers, and nose [30]. As previously mentioned, the movements of these regions serve as clinical assistance for the identification of pain. The evaluation of these changes with the FGS has reported overall inter-rater reliability of (ICC) of 0.89 (95% CI) and intra-rater reliability of 0.91 [31], which denotes the clinical utility of facial expressions. In that regard, facial expressions must be considered when evaluating acute pain in dogs. Pain scales, such as DIVAS (Dynamic Interactive Visual Analogue Scale) and the pain evaluation scales developed by the University of Melbourne (UMPS), the University of Colorado (CSU-CAPS), and the Composed Measure by Glasgow (CMPS), include changes in facial expressions as a part of their assessment. A fixed gaze (Figure 6A), squinting (Figure 6B), or a grimace with a fearful look are examples of this. They also incorporate a glassy appearance of the eyes, mydriasis, furrowed brows, lips drawn back, and flattening of the ears [39,141].

DIVAS includes evaluating changes in facial expression and behaviors and vocalizations derived from pain, such as changes in posture, inactivity, or aggressiveness. A scale from 0 to 100 mm has been used to assess the effects of preemptive analgesia in routine surgeries such as ovariohysterectomy; a patient with a score of 40 mm requires analgesic intervention [148]. Likewise, it has been used to estimate pain intensity in canines undergoing various surgical procedures, such as exploratory laparotomy, hemilaminectomies, osteotomies, and atlantoaxial subluxation [149]. On the other hand, the UMPS considers six categories in which the animal’s appearance includes observations such as the ears’ position and the form of the eyes to classify the changes derived from moderate to severe pain [150].

Finally, the CSU-CAPS considers facial changes such as droopy ears, arched eyebrows, and darting eyes (known as a “worried facial expression”) to assess the level of pain from 0 to 4 (minimal to severe pain), considering a value greater than 2 as a rescue point [151]. Likewise, these parameters have also been proposed as an evaluation method in cats. An example of this is the validated Multidimensional Composite Pain Scale of the University of Botucatu (UNESP-MCPS). In this scale, psychomotor and facial aspects are considered a means of manifestation of pain through changes in facial expression. These include assessing the distance between the ears and the facial muscles’ tension as distinctive features of pain recognition with a sensitivity of 74.6% and specificity of 84.6% [152,153]. This has been used to evaluate postoperative analgesia in procedures such as tibial plateau leveling osteotomies [151] or to determine the perioperative efficacy of opioid drugs such as tramadol during canine sterilization [154].

Nevertheless, all these approaches do not consider specific regions or the distance required to assess the facial expressions and correlate them to the degree of perceived pain. In contrast, in cats, the CMPS-Feline is a scale in which facial evaluation has been included together with behavioral modifications to evaluate postsurgical pain. On a scale from 0 to 20, where 5 or more is considered the analgesic intervention point, facial movements such as the muzzle shape and the ears’ position have been shown to correctly identify 78.6% of animals that required analgesia [155].

For dogs, to date, there are no reports that can associate the intensity of pain to specific facial traits. Therefore, changes in the lips, eyes, and facial muscles are valuable characteristics that can be integrated into the existing pain scales to help identify the intensity and degree of pain in dogs, as done in other species.

Animals cannot communicate pain in a verbal way. Therefore, the study of facial expressions in animals can aid in identifying and differentiating between emotional states such as fear of pain. However, some limitations are present in the implementation of facial changes in pain scales. For example, scales do not consider the residual effects of anesthetics or the sedation level caused by some local analgesics, opioids, sedatives, and cyclohexamines [156]. Additionally, it is important to mention that the accuracy of these scales also depends on the type of pain (i.e., most of the scales have been designed to assess postsurgical pain) [157]. The age, experience, training of the evaluator, and visual acuity when attempting to accurately quantify the degree of pain experienced by a dog based on observations of facial, posture, or behavioral changes can also influence their interpretation [149]. In comparing veterinary students and anesthesiologists, the former gave high scores to those animals that the anesthesiologists rated as low; the study concluded that there was 95% of no agreement between both evaluators [149]. Likewise, the scales are a subjective method that can be vulnerable to evaluator errors or biases. Hence, they are considered an additional tool for the study of pain, since there is no single biomarker, physiological parameters, posture, or facial expression that, by itself, can be considered as the “gold standard” of pain assessment [155].

Further studies in dogs also require the development and implementation of techniques and tools currently applied in other species, such as artificial intelligence and computer vision systems for the recognition of pain gestures in humans and equines [158], or the automatic detection of Facial Units in sheep, a technique with up to 67% accuracy [52]. Likewise, cameras or visual sensors, global positioning systems (GPS), electrocardiography, electroencephalography [159], and infrared thermography [160,161] are alternatives to evaluate emotions and motor responses in dogs comprehensively. It is essential to consider that human perception of emotions can be subjective and that the anatomical characteristics of the dog [162] (e.g., the coat color, the shape of the ears, or the breed itself) can influence and affect the objectivity of facial expression recognition and must be discussed and analyzed before associating facial gestures with a particular emotion.

Today, small species play a particularly important role in the lives of many people by generating a human–animal bond characterized by an acceptance and treatment similar to that of family members [163]. Regarding this, the anatomical differences between breeds represent a challenge for facial expression recognition in dogs. For example, the cranial structural features of prognathic dogs with short muzzles and deep facial folds do not allow adequate recognition of muscle movements and could interfere with the assessment of facial expression in these animals [104]. In contrast, in the case of primitive dogs such as the Siberian or Alaskan Husky breeds, the lack of some facial muscles to perform specific facial movements has been reported [62]. These characteristics can lead to a misinterpretation of negative emotional states such as fear and pain.

Likewise, Bloom et al. [104] have observed that another breed characteristic that intervenes in evaluating the facial expression is the color of the coat. In dogs with black fur, identifying the tension or elevation of certain muscle groups (e.g., supra- or infraorbital) could be a limiting factor. Similarly, small dark spots of hair on the face or in the supraorbital regions lead to a misinterpretation of the meaning of a facial expression [40].

## 9. Conclusions

Facial expressions in animals, including dogs, are a communication form of positive and negative emotions, in which motor responses are developed in specific facial action units. These units include the eyebrows, eyes, position of the ears, opening of the mouth, and others. These movements are the result of neural circuits whose primary structures are found in the limbic system, the amygdala, the motor cortex, and its derivations towards the facial nerve, the main nerve responsible for innervating the muscles of the face and generating the different expressions based on the valence of the stimulus. These expressions are fundamental to maintaining social interaction between dogs and other species, as in relationships with humans.

Although there is evidence of certain patterns of facial changes associated with specific stimuli in domestic dogs, it is challenging to objectively evaluate facial expressions due to domestication, the close human-animal relationship, and the extensive inter-species variability.

Further research in the development of canine facial action scales and the implementation of tools such as computer vision systems, pain scales, infrared thermography, and others are alternatives to evaluate and recognize the association between facial expressions, emotion, and well-being in pets, as well as to use facial expressions as a clinical tool to evaluate pain and diverse emotions in dogs.

## Data Availability

Not applicable.

## References

[1] Crivelli C., Fridlund A.J. (2018). Facial displays are tools for social influence. Trends Cogn. Sci..

[2] Mota-Rojas D., Olmos-Hernández A., Verduzco-Mendoza A., Hernández E., Martínez-Burnes J., Whittaker A. (2020). The utility of grimace escales for practical pain assessment in laboratory animals. Animals.

[3] Darwin C. (1872). The Expressions of the Emotions in Man and Animals.

[4] Boissy A., Manteuffel G., Jensen M.B., Moe R.O., Spruijt B., Keeling L.J., Winckler C., Forkman B., Dimitrov I., Langbein J. (2007). Assessment of positive emotions in animals to improve their welfare. Physiol. Behav..

[5] Konok V., Nagy K., Miklósi Á. (2015). How do humans represent the emotions of dogs? The resemblance between the human representation of the canine and the human affective space. Appl. Anim. Behav. Sci..

[6] Leliveld L.M.C., Langbein J., Puppe B. (2013). The emergence of emotional lateralization: Evidence in non-human vertebrates and implications for farm animals. Appl. Anim. Behav. Sci..

[7] Bennett V., Gourkow N., Mills D.S. (2017). Facial correlates of emotional behaviour in the domestic cat (*Felis catus*). Behav. Proces..

[8] Wathan J., Proops L., Grounds K., McComb K. (2016). Horses discriminate between facial expressions of conspecifics. Sci. Rep..

[9] Wathan J., Burrows A.M., Waller B.M., McComb K. (2015). EquiFACS: The equine facial action coding system. PLoS ONE.

[10] McLennan K.M., Rebelo C.J.B., Corke M.J., Holmes M.A., Leach M.C., Constantino-Casas F. (2016). Development of a facial expression scale using footrot and mastitis as models of pain in sheep. Appl. Anim. Behav. Sci..

[11] Häger C., Biernot S., Buettner M., Glage S., Keubler L.M., Held N., Bleich E.M., Otto K., Müller C.W., Decker S. (2017). The sheep grimace scale as an indicator of post-operative distress and pain in laboratory sheep. PLoS ONE.

[12] Proctor H.S., Carder G. (2014). Can ear postures reliably measure the positive emotional state of cows?. Appl. Anim. Behav. Sci..

[13] Di Giminiani P., Brierley V.L.M.H., Scollo A., Gottardo F., Malcolm E.M., Edwards S.A., Leach M.C. (2016). The assessment of facial expressions in piglets undergoing tail docking and castration: Toward the development of the piglet grimace scale. Front. Vet. Sci..

[14] Viscardi A.V., Hunniford M., Lawlis P., Leach M., Turner P.V. (2017). Development of a piglet grimace scale to evaluate piglet pain using facial expressions following castration and tail docking: A pilot study. Front. Vet. Sci..

[15] Mota-Rojas D., Orihuela A., Martínez-Burnes J., Gómez J., Mora-Medina P., Alavez B., Ramírez L., González-Lozano M. (2020). Neurological modulation of facial expressions in pigs and implications for production. J. Anim. Behav. Biometeorol..

[16] Descovich K. (2017). Facial expression: An under-utilised tool for the assessment of welfare in mammals. ALTEX.

[17] Waller B.M., Micheletta J. (2013). Facial expression in nonhuman animals. Emot. Rev..

[18] Waller B.M., Peirce K., Caeiro C.C., Scheider L., Burrows A.M., McCune S., Kaminski J. (2013). Paedomorphic facial expressions give dogs a selective advantage. PLoS ONE.

[19] Mariti C., Ricci E., Zilocchi M., Gazzano A. (2013). Owners as a secure base for their dogs. Behaviour.

[20] Hare B., Tomasello M. (2005). Human-like social skills in dogs?. Trends Cogn. Sci..

[21] Ford G., Guo K., Mills D. (2019). Human facial expression affects a dog’s response to conflicting directional gestural cues. Behav. Process..

[22] Katayama M., Kubo T., Yamakawa T., Fujiwara K., Nomoto K., Ikeda K., Mogi K., Nagasawa M., Kikusui T. (2019). Emotional contagion from humans to dogs is facilitated by duration of ownership. Front. Psychol..

[23] de Waal F.B.M. (2011). What is an animal emotion?. Ann. N. Y. Acad. Sci..

[24] Ekman P. (1964). Body position, facial expression, and verbal behavior during interviews. J. Abnorm. Soc. Psych..

[25] Bloom T., Friedman H. (2013). Classifying dogs’ (Canis Familiaris) facial expressions from photographs. Behav. Proc..

[26] Reid J., Nolan A.M., Scott E.M. (2018). Measuring pain in dogs and cats using structured behavioural observation. Vet. J..

[27] Corke M.J., Choe C.J. (2019). Indicators of pain. Encyclopedia of Animal Behavior.

[28] Lexis H., Weary D.M. (2021). Facial Expressions in humans as a measure of empathy towards farm animals in pain. PLoS ONE.

[29] McLennan K.M., Miller A.L., Dalla Costa E., Stucke D., Corke M.J., Broom D.M., Leach M.C. (2019). Conceptual and methological issues relating to pain assessment in mammals: The development and ustilisation of pain facial expressions scales. Appl. Anim. Behav. Sci..

[30] Holden E., Calvo G., Collins M., Bell A., Reid J., Scott E.M., Nolan A.M. (2014). Evaluation of facial expression in acute pain in cats. J. Small Anim. Pract..

[31] Evangelista M.C., Watanabe R., Leung V.S.Y., Monteiro B.P., O’Toole E., Pang D.S.J., Steagall P.V. (2019). Facial Expressions of pain in cats: The development and validation of a feline grimace scale. Sci. Rep..

[32] Langford D.J., Bailey A.L., Chanda M.L., Clarke S.E., Drummond T.E., Echols S., Glick S., Ingrao J., Klassen-Ross T., LaCroix-Fralish M.L. (2010). Coding of facial expressions of pain in the laboratory mouse. Nat. Methods.

[33] Sotocinal S.G., Sorge R.E., Zaloum A., Tuttle A.H., Martin L.J., Wieskopf J.S., Mapplebeck J.C., Wei P., Zhan S., Zhang S. (2011). The rat grimace scale: A partially automated method for quantifying pain in the laboratory rat via facial expressions. Mol. Pain.

[34] Lezama-García K., Orihuela A., Olmos-Hernández A., Reyes-Long S., Mota-Rojas D. (2019). Facial expressions and emotions in domestic animals. CAB. Rev..

[35] Ekman P. (1992). Are there basic emotions?. Psychol. Rev..

[36] Harris C., Alvarado N. (2005). Facial expressions, smile types, and self-report during humour, tickle, and pain. Cogn. Emot..

[37] Camerlink I., Coulange E., Farish M., Baxter E.M., Turner S.P. (2018). Facial expression as a potential measure of both intent and emotion. Sci. Rep..

[38] Gibson J.E., Pick D.A. (2000). An Ecological Approach to Perceptual Learning and Development.

[39] Gaynor J.S., Muir W.W. (2015). Handbook of Veterinary Pain Management.

[40] Siniscalchi M., D’Ingeo S., Minunno M., Quaranta A. (2018). Communication in dogs. Animals.

[41] Handelman B. (2012). Canine Behavior: A Photo Illustrated Handbook.

[42] Hecht J., Horowitz A., Jensen P. (2017). Introduction to dog behaviour. The Ethology of Domestic Animals: An Introductory Text.

[43] Kaminski J., Hynds J., Morris P., Waller B.M. (2017). Human attention affects facial expressions in domestic dogs. Sci. Rep..

[44] Kunz M., Faltermeier N., Lautenbacher S. (2012). Impact of visual learning on facial expressions of physical distress: A study on voluntary and evoked expressions of pain in congenitally blind and sighted individuals. Biol. Psychol..

[45] Bremhorst A., Mills D.S., Stolzlechner L., Würbel H., Riemer S. (2021). ´Puppy dog eyes´ are associated with eye movements, not communication. Front. Psychol..

[46] Ferretti V., Papaleo F. (2019). Understanding others: Emotion recognition in humans and other animals. Genes Brain Behav..

[47] Bremhorst A., Sutter N.A., Würbel H., Mills D.S., Riemer S. (2019). Differences in facial expressions during positive anticipation and frustration in dogs awaiting a reward. Sci. Rep..

[48] Panksepp J. (2011). The basic emotional circuits of mammalian brains: Do animals have affective lives?. Neurosci. Biobehav. Rev..

[49] Cano-Vindel A., Fernández E. (1995). Orientaciones en el estudio de la emoción. Manual de Motivación y Emoción.

[50] Domínguez-Oliva A., Mota-Rojas D., Ruiz-García A.G., Miranda-Cortés Á.E., Hernández-Avalos I. (2021). Clinical recognition of stress in dog and cats. AMMVEPE.

[51] Dawson L.C., Cheal J., Niel L., Mason G. (2019). Humans can identify cats’ affective states from subtle facial expressions. Anim. Welf..

[52] Andersen P.H., Broomé S., Rashid M., Lundblad J., Ask K., Li Z., Hernlund E., Rhodin M., Kjellström H. (2021). Towards machine recognition of facial expressions of pain in horses. Animals.

[53] Urrego D., Múnera A., Troncoso J. (2011). Peripheral facial nerve lesion induced long-term dendritic retraction in pyramidal cortico-facial neurons. Biomed. Rev. Inst. Nac. Salud.

[54] Gil V.A. (2008). Anatomía del Sistema Nervioso en el Perro y en el Gato.

[55] Castillo G.D., de Jorge J.L. (2015). Anatomía y Fisiología del Sistema Nervioso Central.

[56] Holmberg J. (1971). The secretory nerves of the parotid gland of the dog. J. Physiol..

[57] Waller B.M., Caeiro C., Peirce K., Burrows A.M., Kaminski J. (2013). DogFACS: The Dog Facial Action Coding System Manual.

[58] Popesko P., Masson S.A. (1998). Atlas de Anatomía Topográfica en los Animales Domésticos.

[59] López P.C., Mayor A.P., Labeaga J.R., López B.M., Pereira T.H.D.S., Monteiro F.O.B. (2018). Atlas de Los Músculos del Perro.

[60] Burrows A.M., Kaminski J., Waller B.M., Omstead K.M., Rogers-Vizena C., Mendelson B. (2021). Dog faces exhibit anatomical differences in comparison to other domestic animals. Anat. Rec..

[61] Vick S.-J., Waller B.M., Parr L.A., Smith Pasqualini M.C., Bard K.A. (2007). A Cross-species comparison of facial morphology and movement in humans and chimpanzees using the Facial Action Coding System (FACS). J. Nonverbal. Behav..

[62] Caeiro C.C., Burrows A., Waller B.M. (2017). Development and application of CatFACS: Are human cat adopters influenced by cat facial expressions?. Appl. Anim. Behav. Sci..

[63] Kaminski J., Waller B.M., Diogo R., Hartstone-Rose A., Burrows A.M. (2019). Evolution of facial muscle anatomy in dogs. Proc. Natl. Acad. Sci. USA.

[64] Sternglanz S.H., Gray J.L., Murakami M. (1977). Adult preferences for infantile facial features: An ethological approach. Anim. Behav..

[65] Archer J., Monton S. (2011). Preferences for infant facial features in pet dogs and cats. Ethology.

[66] Little A.C. (2012). Manipulation of infant-like traits affects perceived cuteness of infant, adult and cat faces. Ethology.

[67] Correira-Caeiro C., Guo K., Mills D.S. (2020). Perception of dynamic facial expressions of emotion between dogs and humans. Anim. Cogn..

[68] Erkoç T., Ağdoğan D., Eskil M.T. (2018). An observation based muscle model for simulation of facial expressions. Signal Process. Image Commun..

[69] Fenton B.W., Shih E., Zolton J. (2015). The neurobiology of pain perception in normal and persistent pain. Pain Manag..

[70] Mota-Rojas D., Mariti C., Zdeinert A., Riggio G., Mora-Medina P., del Mar Reyes A., Gazzano A., Domínguez-Oliva A., Lezama-García K., José-Pérez N. (2021). Anthropomorphism and Its Adverse Effects on the Distress and Welfare of Companion Animals. Animals.

[71] Phillips M.L. (2003). Understanding the neurobiology of emotion perception: Implications for psychiatry. Br. J. Psychiatry.

[72] Calder A.J., Lawrence A.D., Young A.W. (2001). Neuropsychology of fear and loathing. Nat. Rev. Neurosci..

[73] Pertovaara A. (2013). The noradrenergic pain regulation system: A potential target for pain therapy. Eur. J. Pharmacol..

[74] Reid K., Rogers C.W., Gronqvist G., Gee E.K., Bolwell C.F. (2017). Anxiety and pain in horses measured by heart rate variability and behavior. J. Vet. Behav..

[75] Hu J., Qi S., Becker B., Luo L., Gao S., Gong Q., Hurlemann R., Kendrick K.M. (2015). Oxytocin selectively facilitates learning with social feedback and increases activity and functional connectivity in emotional memory and reward processing regions. Hum. Brain Mapp..

[76] Valenchon M., Lévy F., Moussu C., Lansade L. (2017). Stress Affects instrumental learning based on positive or negative reinforcement in interaction with personality in domestic horses. PLoS ONE.

[77] Powell L., Guastella A.J., McGreevy P., Bauman A., Edwards K.M., Stamatakis E. (2019). The physiological function of oxytocin in humans and its acute response to human-dog interactions: A review of the literature. J. Vet. Behav..

[78] Alexander R., Aragón O.R., Bookwala J., Cherbuin N., Gatt J.M., Kahrilas I.J., Kästner N., Lawrence A., Lowe L., Morrison R.G. (2021). The neuroscience of positive emotions and affect: Implications for cultivating happiness and wellbeing. Neurosci. Biobehav. Rev..

[79] Osella M., Odore R., Badino P., Cuniberti B., Bergamasco L. (2005). Plasma dopamine neurophysiological correlates in anxious dogs. Current Issues and Research in Veterinary Behavioral Medicine.

[80] Karpiński M., Ognik K., Garbiec A., Czyżowski P., Krauze M. (2021). Effect of stroking on serotonin, noradrenaline, and cortisol levels in the blood of right- and left-pawed dogs. Animals.

[81] Siracusa C., Denenberg S. (2021). IAggression–Dogs. Small Animal Veterinary Psychiatry.

[82] Bethlehem R.A.I., van Honk J., Auyeung B., Baron-Cohen S. (2013). Oxytocin, brain physiology, and functional connectivity: A review of intranasal oxytocin FMRI studies. Psychoneuroendocrinology.

[83] Domes G., Heinrichs M., Gläscher J., Büchel C., Braus D.F., Herpertz S.C. (2007). Oxytocin attenuates amygdala responses to emotional faces regardless of valence. Biol. Psychiatry.

[84] Petersson M., Uvnäs-Moberg K., Nilsson A., Gustafson L.L., Hydbring-Sandberg E., Handlin L. (2017). Oxytocin and cortisol levels in dog owners and their dogs are associated with behavioral patterns: An exploratory study. Front. Psychol..

[85] Mitsui S., Yamamoto M., Nagasawa M., Mogi K., Kikusui T., Ohtani N., Ohta M. (2011). Urinary oxytocin as a noninvasive biomarker of positive emotion in dogs. Horm. Behav..

[86] Lansade L., Nowak R., Lainé A.-L., Leterrier C., Bonneau C., Parias C., Bertin A. (2018). facial expression and oxytocin as possible markers of positive emotions in horses. Sci. Rep..

[87] Ogi A., Mariti C., Baragli P., Sergi V., Gazzano A. (2020). Effects of stroking on salivary oxytocin and cortisol in guide dogs: Preliminary results. Animals.

[88] MacLean E.L., Gesquiere L.R., Gee N.R., Levy K., Martin W.L., Carter C.S. (2017). Effects of affiliative human-animal interaction on dog salivary and plasma oxytocin and vasopressin. Front. Psychol..

[89] Somppi S., Törnqvist H., Topál J., Koskela A., Hänninen L., Krause C.M., Vainio O. (2017). Nasal oxytocin treatment biases dogs’ visual attention and emotional response toward positive human facial expressions. Front. Psychol..

[90] Mobbs D. (2018). What Can the social emotions of dogs teach us about human emotions?. Anim. Sentience.

[91] Anderson D.J., Adolphs R. (2014). A Framework for studying emotions across species. Cell.

[92] Lindsley D.B., Stevens S.S. (2018). Emotion. Handbook of Experimental Psychology.

[93] Nummenmaa L., Saarimäki H. (2019). Emotions as discrete patterns of systemic activity. Neurosci. Lett..

[94] Hess U., Hareli S. (2015). The role of social context for the interpretation of emotional facial expressions. Understanding Facial Expressions in Communication.

[95] Kraaijenvanger E.J., Hofman D., Bos P.A. (2017). A neuroendocrine account of facial mimicry and its dynamic modulation. Neurosci. Biobehav. Rev..

[96] Brudzynski S.M. (2010). Communication of emotions in animals. Encyclopedia of Behavioral Neuroscience.

[97] Nagasawa M., Murai K., Mogi K., Kikusui T. (2011). Dogs can discriminate human smiling faces from blank expressions. Anim. Cogn..

[98] Albuquerque N., Guo K., Wilkinson A., Resende B., Mills D.S. (2018). Mouth-licking by dogs as a response to emotional stimuli. Behav. Proc..

[99] Karl S., Sladky R., Lamm C., Huber L. (2021). Neural responses of pet dogs witnessing their caregiver´s positive interactions with a conspecific: An fMRI study. Cereb. Cortex Commun..

[100] Kujala M., Somppi S., Jokela M., Vainio O., Parkkonen L. (2017). Human empathy, personality and experience affect the emotion ratings of dog and human facial expressions. PLoS ONE.

[101] Beerda B., Schilder M.B.H., van Hooff J.A.R.A.M., de Vries H.W. (1997). Manifestations of chronic and acute stress in dogs. Appl. Anim. Behav. Sci..

[102] Caeiro C., Guo K., Mills D. (2017). Dogs and humans respond to emotionally competent stimuli by producing different facial actions. Sci. Rep..

[103] Meints K., Racca A., Hickey N. (2010). How to prevent dog bite injuries? Children misinterpret dogs, facial expressions. Proceedings of the 10th World Conference on Injury Prevention and Safety Promotion.

[104] Bloom T., Trevathan-Minnis M., Atlas N., MacDonald D.A., Friedman H.L. (2021). Identifying facial expressions in dogs: A replication and extension study. Behav. Proc..

[105] Boissy A., Dwyer C.M., Jones R.B. (2011). Fear and other negative emotions. Animal Welfare.

[106] Flint H.E., Coe J.B., Pearl D.L., Serpell J.A., Niel L. (2018). Effect of training for dog fear identification on dog owner ratings of fear in familiar and unfamiliar dogs. Appl. Anim. Behav. Sci..

[107] Mariti C., Gazzano A., Moore J.L., Baragli P., Chelli L., Sighieri C. (2012). Perception of dogs´stress their owners. J. Vet. Behav..

[108] Racca A., Amadei E., Ligout S., Guo K., Meints K., Mills D. (2010). Discrimination of human and dog faces and inversion responses in domestic dogs (Canis familiaris). Anim. Cogn..

[109] Racca A., Guo K., Meints K., Mills D.S. (2012). Reading faces: Differential Lateral gaze bias in processing canine and human facial expressions in dogs and 4-year-old children. PLoS ONE.

[110] Stetina B.U., Turner K., Burger E., Glenk L.M., McElheney J.C., Handlos U., Kothgassner O.D. (2011). Learning emotion recognition from canines? Two for the road. J. Vet. Behav..

[111] Ogura T., Maki M., Nagata S., Nakamura S. (2020). Dogs (Canis Familiaris) gaze at our hands: A preliminary eye-tracker experiment on selective attention in dogs. Animals.

[112] Travain T., Valsecchi P. (2021). Infrared thermography in the study of animals´emotional responses: A critical review. Animals.

[113] Langford D.J. (2006). Social modulation of pain as evidence for empathy in mice. Science.

[114] Jeon D., Kim S., Chetana M., Jo D., Ruley H.E., Lin S.-Y., Rabah D., Kinet J.-P., Shin H.-S. (2010). Observational fear learning involves affective pain system and Cav1.2 Ca^2+^ channels in ACC. Nat. Neurosci..

[115] Hess U., Fischer A. (2013). Emotional mimicry as social regulation. Personal. Soc. Psychol. Rev..

[116] Ha J.C., Campion T.L. (2019). The emotional animal: Using the science of emotions to interpret behavior. Dog Behavior.

[117] Kurachi T., Irimajiri M., Mizuta Y., Satoh T. (2017). Dogs predisposed to anxiety disorders and related factors in japan. Appl. Anim. Behav. Sci..

[118] Van Bourg J., Patterson J.E., Wynne C.D.L. (2020). Pet dogs (Canis Lupus Familiaris) release their trapped and distressed owners: Individual variation and evidence of emotional contagion. PLoS ONE.

[119] Marcet-Rius M., Pageat P., Bienboire-Frosini C., Teruel E., Monneret P., Leclercq J., Lafont-Lecuelle C., Cozzi A. (2018). Tail and ear movements as possible indicators of emotions in pigs. Appl. Anim. Behav. Sci..

[120] Reefmann N., Bütikofer Kaszàs F., Wechsler B., Gygax L. (2009). Ear and tail postures as indicators of emotional valence in sheep. Appl. Anim. Behav. Sci..

[121] Briefer Freymond S., Briefer E.F., Zollinger A., Gindrat-von Allmen Y., Wyss C., Bachmann I. (2014). Behaviour of horses in a judgment bias test associated with positive or negative reinforcement. Appl. Anim. Behav. Sci..

[122] Arena L., Wemelsfelder F., Messori S., Ferri N., Barnard S. (2017). Application of free choice profiling to assess the emotional state of dogs housed in shelter environments. Appl. Anim. Behav. Sci..

[123] Tami G., Gallagher A. (2009). Description of the behaviour of domestic dog (Canis Familiaris) by experienced and inexperienced people. Appl. Anim. Behav. Sci..

[124] Diesel G., Brodbelt D., Pfeiffer U.D. (2008). Reliability of assessment of dogs´behavioural responses by staff working at a welfare charuty in the UK. Appl. Anim. Behav. Sci..

[125] Lakestani N.N., Donaldson M.L., Waran N. (2014). Interpretation of dog behavior by children and young adults. Anthrozoös.

[126] Merola I., Prato-Previde E., Marshall-Pescini S. (2012). Dogs’ social referencing towards owners and strangers. PLoS ONE.

[127] Horowitz A. (2009). Disambiguating the “Guilty Look”: Salient prompts to a familiar dog behaviour. Behav. Proc..

[128] Kujala M. (2017). Canine Emotions as seen through human social cognition. Anim. Sentience.

[129] Correia-Caeiro C., Guo K., Mills D. (2021). Bodily emotional expressions are a primary source of information for dogs, but not for humans. Anim. Cogn..

[130] Csoltova E., Mehinagic E. (2020). Where do we stand in the domestic dog (Canis familiaris) positive-emotion assessment: A state-of-the-art review and future directions. Front. Psychol..

[131] Bremhorst A., Bütler S., Würbel H., Riemer S. (2018). Incentive motivation in pet dogs–Preference for constant vs varied food rewards. Sci. Rep..

[132] Siniscalchi M., d’Ingeo S., Quaranta A. (2018). Orienting asymmetries and physiological reactivity in dogs’ response to human emotional faces. Learn. Behav..

[133] Firnkes A., Bartels A., Bidoli E., Erhard M. (2017). Appeasement signals used by dogs during dog–human communication. J. Vet. Behav..

[134] Nagasawa M., Kawai E., Mogi K., Kikusui T. (2013). Dogs show left facial lateralization upon reunion with their owners. Behav. Proc..

[135] Siniscalchi M., D’Ingeo S., Fornelli S., Quaranta A. (2017). Are dogs red–green colour blind?. R. Soc. Open Sci..

[136] Raja S.N., Carr D.B., Cohen M., Finnerup N.B., Flor H., Gibson S., Keefe F.J., Mogil J.S., Ringkamp M., Sluka K.A. (2020). The revised international association for the study of pain definition of pain: Concepts, challenges, and compromises. Pain.

[137] Mota-Rojas D., Orihuela A., Strappini-Asteggiano A., Nelly Cajiao-Pachón M., Agüera-Buendía E., Mora-Medina P., Ghezzi M., Alonso-Spilsbury M. (2018). Teaching Animal Welfare in Veterinary Schools in Latin America. Int. J. Vet. Sci. Med..

[138] Hernandez-Avalos I., Mota-Rojas D., Mora-Medina P., Martínez-Burnes J., Casas-Alvarado A., Verduzco-Mendoza A., Lezama-García K., Olmos-Hernandez A. (2019). Review of different methods used for clinical recognition and assessment of pain in dogs and cats. Int. J. Vet. Sci. Med..

[139] de Grauw J.C., van Loon J.P.A.M. (2016). Systematic pain assessment in horses. Vet. J..

[140] Gleerup K.B., Forkman B., Lindegaard C., Andersen P.H. (2015). An equine pain face. Vet. Anaesth. Anal..

[141] Hernández-Avalos I., Mota Rojas D., Mendoza-Flores J.E., Casas-Alvarado A., Flores-Padilla K., Miranda-Cortes A.E., Torres-Bernal F., Gómez-Prado J., Mora-Medina P. (2021). Nociceptive pain and anxiety in equines: Physiological and behavioral alterations. Vet. World.

[142] Dalla Costa E., Stucke D., Dai F., Minero M., Leach M., Lebelt D. (2016). Using the horse grimace scale (HGS) to assess pain associated with acute laminitis in horses (*Equus caballus*). Animals.

[143] Parr L.A., Waller B.M., Burrows A.M., Gothard K.M., Vick S.J. (2010). Brief communication: MaqFACS: A muscle-based facial movement coding system for the rhesus macaque. Am. J. Phys. Anthropol..

[144] Keating S.C.J., Thomas A.A., Flecknell P.A., Leach M.C. (2012). Evaluation of EMLA cream for preventing pain during tattooing of rabbits: Changes in physiological, behavioural and facial expression responses. PLoS ONE.

[145] Reijgwart M.L., Schoemaker N.J., Pascuzzo R., Leach M.C., Stodel M., de Nies L., Hendriksen C.F.M., van der Meer M., Vinke C.M., van Zeeland Y.R.A. (2017). The composition and initial evaluation of a grimace scale in ferrets after surgical implantation of a telemetry probe. PLoS ONE.

[146] Camps T., Amat M., Mariotti V.M., Le Brech S., Manteca X. (2012). Pain-related aggression in dogs: 12 clinical cases. J. Vet. Behav..

[147] Dalla Costa E., Minero M., Lebelt D., Stucke D., Canali E., Leach M.C. (2014). Development of the horse grimace scale (HGS) as a pain assessment tool in horses undergoing routine castration. PLoS ONE.

[148] Niella R.V., Sena A.S., Corrêa J.M.X., Soares P.C.L.R., Pinto T.M., Silva Junior A.C., Costa B.A., de Oliveira J.N.S., da Silva E.B., de Lavor M.S.L. (2020). Preemptive effect of amantadine as adjuvant in postoperative analgesia of ovaryhisterectomy in dogs. Res. Soc. Dev..

[149] Barletta M., Young C.N., Quandt J.E., Hofmeister E.H. (2016). Agreement between veterinary students and anesthesiologists regarding postoperative pain assessment in dogs. Vet. Anaesth. Analg..

[150] Kinjavdekar S.P., Aithal A.H.P., Pawde A.M., Malik V. (2010). Comparison of analgesic effects of meloxicam and ketoprofen using university of melbourne pain scale in clinical canine orthopaedic patients. J. Appl. Anim. Res..

[151] Reader R.C., McCarthy R.J., Schultz K.L., Volturo A.R., Barton B.A., O’Hara M.J., Abelson A.L. (2020). Comparison of liposomal bupivacaine and 0.5% bupivacaine hydrochloride for control of postoperative pain in dogs undergoing tibial plateau leveling osteotomy. J. Am. Vet. Med. Assoc..

[152] Brondani J.T., Mama K.R., Luna S.P.L., Wright B.D., Niyom S., Ambrosio J., Vogel P.R., Padovani C.R. (2013). Validation of the English Version of the UNESP-Botucatu Multidimensional Composite Pain Scale for Assessing Postoperative Pain in Cats. BMC Vet. Res..

[153] Belli M., de Oliveira A.R., de Lima M.T., Trindade P.H.E., Steagall P.V., Luna S.P.L. (2021). Clinical validation of the Short and Long UNESP-Botucatu Scales for Feline Pain Assessment. PeerJ.

[154] Meunier N.V., Panti A., Mazeri S., Fernandes K.A., Handel I.G., Bronsvoort B.M.d.C., Gamble L., Mellanby R.J. (2019). Randomised trial of perioperative tramadol for canine sterilisation pain management. Vet. Rec..

[155] Reid J., Scott E.M., Calvo G., Nolan A.M. (2017). Definitive Glasgow acute pain scale for cats: Validation and intervention level. Vet. Rec..

[156] Steagall P.V., Beatriz M.P. (2019). Acute pain in cats recent advances in clinical assessment. J. Feline Med. Surg..

[157] Mich P.M., Hellyer P.W., Gaynor J.S., Muir W.W. (2008). Objective, categoric methods for assessing pain and analgesia. Handbook of Veterinary Pain Management.

[158] Lu Y., Mahmoud M., Robinson P. Estimating sheep pain level using facial action unit detection. Proceedings of the 12th IEEE International Conference on Automatic Face and Gesture Recognition.

[159] Neethirajan S., Reimert I., Kemp B. (2021). Measuring farm animal emotions—Sensor-based approaches. Sensors.

[160] Casas-Alvarado A., Mota-Rojas D., Hernández-Avalos I., Mora-Medina P., Olmos-Hernández A., Verduzco-Mendoza A., Reyes-Sotelo B., Martínez-Burnes J. (2020). Advances in infrared thermography: Surgical aspects, vascular changes, and pain monitoring in veterinary medicine. J. Therm. Biol..

[161] Mota-Rojas D., Pereira A., Wang D., Martínez-Burnes J., Ghezzi M., Lendez P., Bertoni A., Geraldo A.M. (2021). Clinical applications and factors involved in validating thermal windows used in infrared thermography to assess health and productivity. Animals.

[162] Reyes-Sotelo B., Mota-Rojas D., Martínez-Burnes J., Gómez J., Lezama K., González-Lozano M., Hernández-Ávalos I., Casas A., Herrera Y., Mora-Medina P. (2020). Tail docking in dogs: Behavioural, physiological and ethical aspects. CAB Rev..

[163] Mota-Rojas D., Broom D.M., Orihuela A., Velarde A., Napolitano F., Alonso-Spilsbury M. (2020). Effects of human-animal relationship on animal welfare. J. Anim. Behav. Biometeorol..

